# An Investigation of the Intermolecular Interactions and Recognition Properties of Molecular Imprinted Polymers for Deltamethrin through Computational Strategies

**DOI:** 10.3390/polym11111872

**Published:** 2019-11-13

**Authors:** Lei Xie, Nan Xiao, Lu Li, Xinan Xie, Yan Li

**Affiliations:** College of Food Science, South China Agricultural University, No. 483, Wushan Street, Tianhe District, Guangzhou 510642, China; xielei@stu.scau.edu.cn (L.X.); xiaonan@scau.edu.cn (N.X.); xinanxie@scau.edu.cn (X.X.); yanli@scau.edu.cn (Y.L.)

**Keywords:** density functional theory, deltamethrin, acrylamide, molecular imprinted polymer, intermolecular interaction, recognition property

## Abstract

Deltamethrin (DM) is a toxic pesticide that is nonetheless widely used to control insect pests in agricultural production. Although the number of DM molecularly imprinted polymers (MIPs) is increasing in many scientific applications, the theoretical aspects of the participating intramolecular forces are not fully understood. This paper aims to explore the intermolecular interactions between the template molecule DM and the functional monomer acrylamide (AM) through density functional theory (DFT), analysis of hydrogen nuclear magnetic resonance (^1^H-NMR), Fourier transform infrared spectroscopy (FTIR), and adsorption thermodynamics. The results indicated that there is strong hydrogen bonding between O19 of DM and H9 of AM, suggesting that it is the preferable site for the binding of the target molecule. The existence of interaction sites was found to play an important role in the recognition process. The results from selective adsorption experiments showed that the DM MIPs exhibited the highest adsorption capacity for DM (Q = 75.72 mg g^−1^) as compared to the five structural analogs. Furthermore, the recovery rates of spiked DM from various teas using the DM MIPs as solid-phase extraction filler also possessed a high value (all greater than 83.68%), which enables them to be used as separate and recognition functional materials.

## 1. Introduction

In recent years, the use of pyrethroid pesticides in agricultural production and public health-related areas has increased significantly owing to their broad spectrum and high efficiency [1]. Deltamethrin (DM) is a synthetic pyrethroid and is employed extensively to control insect pests in tea plantations. However, it exhibits high toxicity and has an adverse effect on the central nervous system of humans as well as the aquatic environment [2]. Therefore, its presence can be a major problem with respect to food safety. This has made it necessary to develop robust analytical methods for its detection with high precision and selectivity.

Molecular imprinting technology is an advanced technique that can be used to create molecular imprinted polymers (MIPs) with tailor-made binding sites that are complementary to the shape, size, and functional groups of the template molecule [3]. MIPs show remarkable template recognition properties and have been used successfully for the detection and removal of DM from food samples [4,5,6]. Studies have shown that the recognition properties and the physical properties of MIPs are dependent on the success of template–monomer interactions during the prepolymerization stage [7,8,9]. Therefore, various analytical techniques, including Fourier transform infrared spectroscopy (FTIR) and nuclear magnetic resonance (NMR) spectroscopy, have been used to analyze the intermolecular interactions between the template and the monomers in the prepolymerization complex [10,11,12]. Even so, studies on the number and relative positions of interaction sites between template molecules and functional monomer are still lacking, which may influence the imprinted efficiency of MIPs.

With the rapid development of computer technology, computer simulations have emerged as an efficient mean to assist the design of MIPs in determining the optimal functional monomer, optimizing the synthesis conditions and obtaining additional information of polymerization process [13,14,15,16]. Our previous study also used the methods of computer molecular dynamics and ultraviolet spectroscopy to screen functional monomers and solvents suitable for DM templates, and successfully synthesized DM MIPs with high specificity and selectivity [17]. However, molecular simulation technology has not only been used to identify the optimal synthesis conditions for designing MIPs, but also to detect molecular structure and intermolecular interactions with great accuracy, thus providing useful insights into the recognition mechanism of MIPs. Density functional theory (DFT), a quantum chemistry calculation method, is commonly used for the rapid and accurate calculation of the molecular structures and properties of the compounds involved in an MIPs system [18,19]. For instance, Liu et al. successfully predicted the hydrogen bonding sites and hence the binding locations in the hydroxyflavone conformation based on DFT calculations [20]. Khan et al. analyzed the polymeric precursors, natural orbital charge, and molecular electrostatic potential of a tetrachlorodibenzo-p-dioxin MIPs and performed a benchmark study on the binding energy for dioxin-imprinted polymer complexes [21]. From a practical perspective, simulations of the frontier molecular orbitals (FMOs), molecular electrostatic potentials (MEPs), and Fukui functions can enhance our understanding of the interactions that occur between the template and functional monomer. However, reports of DFT simulation involving the interaction of template molecule DM-functional monomer acrylamide (AM) prepolymerization systems are uncommon.

In the present study, we elucidated the recognition sites of DM-AM prepolymerization system through DFT calculations of the MEPs, FMOs, and Fukui functions of the template and monomer. We validated the DFT model by comparing the simulation results with the experimentally obtained data. Moreover, thermodynamic analysis and selective adsorption experiments were performed to evaluate the recognition characteristics of the DM MIPs. The results of this work provide a more detailed view of the intermolecular interactions that occur between DM molecules and their functional counterparts and lays the foundation for the efficient synthesis of effective DM MIPs.

## 2. Materials and Methods

### 2.1. Materials

DM, cypermethrin, fenvalerate, fenpropathrin, lambda-cyhalothrin, and bifenthrin (>98% purify) samples were purchased from Jiangsu Rongde Reagent Factory (Zhenjiang, China). Green tea, black tea, and tieguanyin (dried tea) were purchased from Fujian Anxi Country Xiyun Tea Industry Co., Ltd. (Quanzhou, China). Black tea beveragewas purchased from Nongfu Spring Jiande New Anjiang Beverage Co., Ltd. (Hangzhou, China). Furthermore, AM, ethylene glycol dimethacrylate (EGDMA), and azobisisobutyronitrile (AIBN) were bought from Aladdin Industrial Corporation (Shanghai, China). Absolute ethanol and acetic acid were obtained from Guangdong Guanghua Technology Co., Ltd. (Shantou, China). Methanol was purchased from Guangzhou Dongjiang Chemical Plant (Guangzhou, China). Acetone-D6 was purchased from Merck Chemical Technology Co., Ltd. (Shanghai, China). Sodium lauryl sulfate were purchased from Shanghai Lingfeng Chemical Reagent Co., Ltd. (Shanghai China). Dibutyl phthalate was purchased from Tianjin Bodi Chemical Co., Ltd. (Tianjin China). Hydroxyethyl cellulose (HEC) was purchased from Guangzhou Qingfeng Chemical Co., Ltd. (Guangzhou China). Chloroform, acetonitrile, and octanol were purchased from Tianjin Damao Chemical Reagent Factory (Tianjin China). N-hexane and acetone were purchased from Guangdong Guanghua Technology Co., Ltd. (Shantou, China).

### 2.2. Computational Methods and Characterization

The DMol3 of Materials Studio (Accelrys, Sortware, Inc., San Diego, CA, USA) was used, which is widely used for theoretical studies [22,23]. We performed geometric optimization and calculations of molecular structure of DM and functional monomer AM. The exchange correlation effects were accounted for by using the generalized gradient approximation (GGA) of the BLYP functional. The double numerical plus polarization basis set, were used to ensure highly accurate results. The nuclear treatment method used was election relativistic. Based on the abovementioned basis set, we analyzed the MEPs, FMOs, and Fukui functions of DM and AM.

The molecular structure model of the template molecule DM and the functional monomer AM was established by the GaussView 5.0.9 program (Gaussian, Inc., Wallingford, CT, USA). Based on the DFT method, the density-based solvation model (SMD) model of B3LYP/6-31G + (d, p) level was used to calculate the solvation energy values of the template and monomer in four different solvents, and the thermodynamic parameters of AM in a chloroform solvent environment were also analyzed. In order to obtain a more accurate Gibbs free energy (ΔG), enthalpy (ΔH) and entropy (ΔS), all frequencies were corrected using a correction factor (f = 0.9613) [24] during the calculation.

### 2.3. H-NMR Analysis

Acetone-D6 was used as the solvent to prepare approximately 5 mmol L^−1^ of a solution of the functional monomer (AM) and functional template (AM-DM), and the solutions were subsequently sonicated for 5 min. Next, after being left to stand overnight, the solution was analyzed using a Bruker AMX 600 MHz spectrometer (Bruker, Karlsruhe, Germany) at room temperature to obtain its ^1^H-NMR spectrum, which was used to evaluate the interactions between DM and AM in the prepolymerization mixture.

### 2.4. FTIR Analysis

A DM/AM mixture with a molar ratio of 1:6 was prepared in chloroform. The DM-AM complex was prepared by stirring in a 65 °C water bath for 6 h, followed by drying in an oven at 65 °C for 6 h. Next, 0.001 g of a DM–AM complex, 0.001 g of DM, and 0.001 g of AM were used to prepare the KBr pellet samples used for the FTIR spectral measurements (Vertex 70, Beijing, China). All the FTIR measurements were carried out at room temperature and were performed for wavenumbers of 500 to 4000 cm^−1^.

### 2.5. Thermodynamic Properties

The thermodynamic parameters Δ*G*, Δ*H*, and Δ*S* (Equations (1), (2), and (5)) of AM were calculated using Gaussian simulation software (Gaussian, Inc., Wallingford, CT, USA). In addition, in order to evaluate the spontaneity of the synthesized DM MIPs for the DM adsorption process, the thermodynamic parameters can be calculated using the following equations (Equations (3)–(5)):

(1) $$\Delta G=\varepsilon_{0}+G_{corr}$$

(2) $$\Delta H=\varepsilon_{0}+H_{corr}$$

(3) $$InC_{e}=InK_{}+\frac{\Delta H}{\mathrm{RT}}$$

(4) ΔG=−nRT

(5) $$\Delta S=\frac{\Delta H-\Delta G}{T},$$

where ε_0_ is the total electronic energy, *G*_corr_ is the thermal free energy correction, *H*_corr_ is the enthalpy correction (zero-point energy), *C*_e_ (μmol mL^−1^) is the equilibrium concentration, *T* (K) is the absolute temperature, R (8.314 J mol^−1^ K^−1^) is the ideal gas constant, n is an empirically determined parameter in the Freundlich model, and K is a constant.

### 2.6. Solvation Analysis

The SMD model was selected to optimize the molecular configuration and calculate its energy. The difference in configuration energy between the gas phase and the solution was used as the solvation energy, which can indicate the influence of the solvent environment on the molecular system. The solvation energy values of the template and monomer in four different solvents were calculated using Equation (6):

(6) $$E_{in\;solvation}=E_{in\;gas}-E_{in\;solvent},$$

where *E*_in solvation_ is the solvation energy of the system, *E*_in gas_ is the single point energy in a gas phase environment, and *E*_in solvent_ is the single point energy for solvent conditions.

### 2.7. Synthesis of DM MIPs

The DM MIPs were prepared by a previously reported two-step seed swelling polymerization process [25]. In brief, first, a known amount of polystyrene seed spheres and 0.2 g of sodium dodecyl sulfate were added to 100 mL of water and dispersed by ultrasonication. The mixture was then transferred to a four-necked flask. Another solution was prepared by adding 0.1 g of AIBN and 1 mL of dibutyl phthalate to 75 mL of water and adding the mixture to the flask. The two solutions were mixed by stirring. Next, 1 mmoL of DM, 6 mmol of AM, 20 mmoL of EGDMA, 0.4 g of HEC, 175 mL of water, 5 mL of octanol, and 10 mL of chloroform were added into the solution; the droplet size was less than 0.5 µm. This was followed by continuous stirring. Finally, 1.2 g of HEC and 50 mL of water were added to the solution, and polymerization was performed at 70 °C for 24 h. The obtained product was washed and dried under a vacuum. Subsequently, the template molecules were removed by extraction, and the DM MIPs were obtained after drying at 60 °C for 24 h. Nonimprinted polymers (NIPs) were also prepared using a similar process but without the molecular template.

### 2.8. Evaluation of Specific Adsorption Characteristics

First, 25 mg of the MIPs were added to an Erlenmeyer flask containing 25 mL of DM and its structural analogues (cypermethrin, fenvalerate, fenpropathrin, lambda-cyhalothrin, and bifenthrin) at an initial concentration of 100 mg L^−1^. The flask was shaken at 303 K for 24 h, after which the polymer was removed by filtration, and the absorbance of the liquid was determined at wavelengths specific to each pesticide using an ultraviolet-visible spectrophotometer. The adsorption capacity (Q) was determined using Equation (7):

(7) $$Q=\frac{V(C_{s0}-C_{s})}{M},$$

where *C*_S_ is the concentration of the analyte in the solution once adsorption equilibration had been reached (mg L^−1^), *C*_S0_ is the initial concentration (mg L^−1^), *M* is the mass of the polymer (g), and *V* is the volume of the solution (mL).

### 2.9. The Adsorption of DM in Different Teas by DM MIPs Solid-Phase Extraction

DM MIPs was used as a solid phase extraction column filler to study its adsorption properties of DM in different teas (see the Appendix A for the methodology).

The recovery rate of DM via solid phase extraction can be calculated using Equation (8):

(8) $$\mathrm{The}\;\mathrm{recovery}\;\mathrm{rate}\;\mathrm{of}\;\mathrm{DM}=\frac{C_{s}}{C_{s0}}\times 100,$$

where *C*_S_ is the concentration of the analyte in the eluent and *C*_S0_ is the initial concentration.

## 3. Results and Discussion

### 3.1. Active Sites for DM and AM Binding

#### 3.1.1. Analysis of Frontier Molecular Orbitals

**Figure 1:**
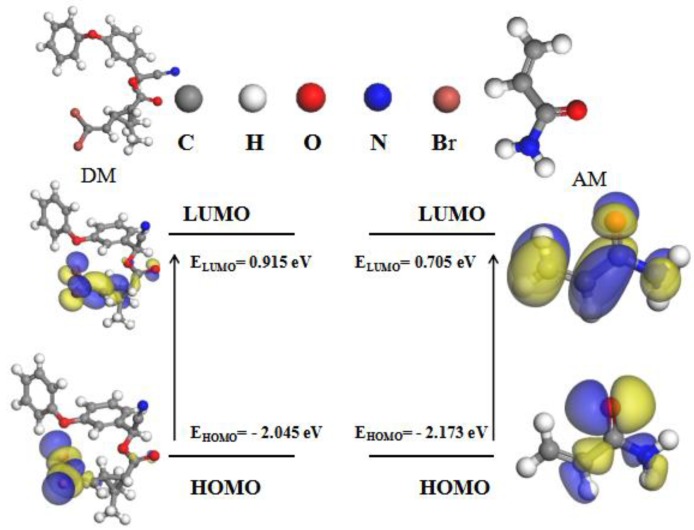
Frontier molecular orbitals and energy levels for HOMO and LUMO of DM and AM.

The FMO analysis method is efficient for studying intra- and intermolecular binding as well as the interactions between bonds. It also allows one to investigate the charge transfer or conjugative interactions within molecular systems [26]. The delocalization of the electron density between the occupied and formally unoccupied FMOs is indicative of stable donor–acceptor interactions, suggesting that the atoms in the highest occupied molecular orbital (HOMO) readily provide electrons, while the lowest unoccupied molecular orbital (LUMO) readily receives them [27]. To predict the imprinted active sites for DM and AM binding, the FMO analysis results for DM and AM were plotted (Figure 1).

As shown in Figure 1, in the template molecule, the –C=O group occupies the part of LUMO. In the case of the functional monomer, AM, –C=O, and –NH– occupy both HOMO and LUMO orbitals, indicating that AM may act as both an electron donor and an electron acceptor. However, the frontier orbital theory based on quantum mechanics holds that the lower the energy of the LUMO (*E*_LUMO_), the greater the capacity to accept electrons will be. By contrast, the higher the energy of the HOMO (*E*_HOMO_), the greater the capacity to donate electrons will be. Therefore, it can be seen from the analysis results that E_LUMO_ value of DM is 0.915 eV, which is higher than that of AM; this confirmed that DM was the main electron donor. The E_HOMO_ value of AM is −2.173 eV, which is lower than that of DM, indicating that AM is the main electron accepter. Thus, DM can easily donate electrons, and as such possesses higher reactivity than AM.

#### 3.1.2. Analysis of Molecular Electrostatic Potentials

**Figure 2:**
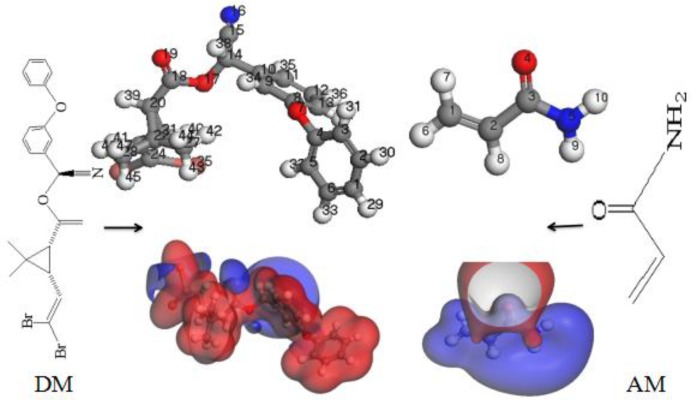
Molecular electrostatic potentials of DM and AM.

The MEP, which is related to the electron density, is critical for studying the molecular imprinted active sites, the recognition process, and the formation of hydrogen bonds [28,29]. Therefore, we calculated the electrostatic potentials and distributions of the electron clouds of DM and AM; the MEP is shown in Figure 2.

Based on the distribution of the electron cloud, the active sites could be directly predicted. Blue indicates the electron-rich region, where the electrostatic potential is negative. In contrast, the red region is the electron-deficient region, where the electrostatic potential is positive. As can be seen in Figure 2, atoms O4, N5, and H9 of the AM characteristic group are positively charged, indicating that it is at a lower energy level and can be easily attacked by nucleophiles and hence made to accept electrons. Thus, they usually act as electron acceptors. On the other hand, atom H10 of AM is negatively charged, indicating that it is at a higher energy level and hence susceptible to attack by electrophiles (and can lose electrons). It is thus an electron donor. For the DM characteristic group, atom O19 on the carbonyl group is an electron donor in the electron-rich region, and atom O17 is an electron acceptor. Therefore, based on the results of the analysis of the FMOs, the active sites were speculated to be atoms O4, N5, and H9 for AM and O19 for DM.

#### 3.1.3. Analysis of Fukui Functions

**Figure 3:**
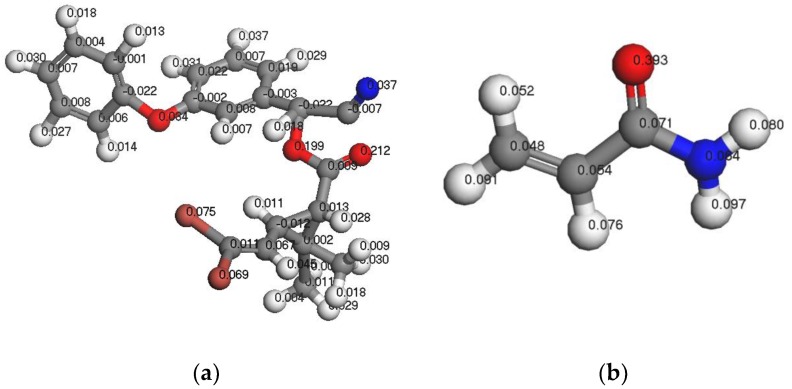
F (−) functions of DM (**a**) and AM (**b**).

**Figure 4:**
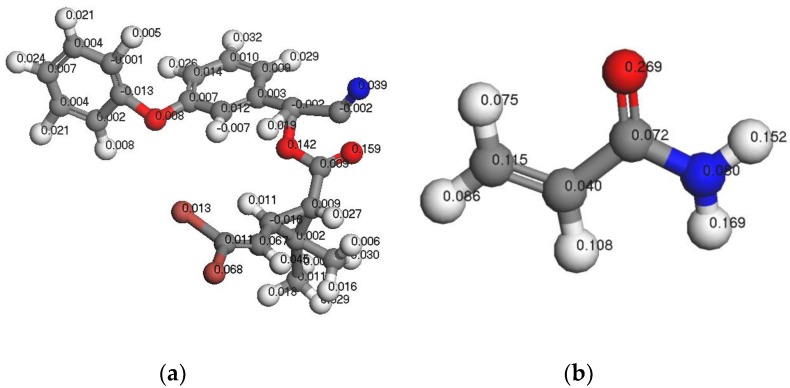
F (0) functions of DM (**a**) and AM (**b**).

**Figure 5:**
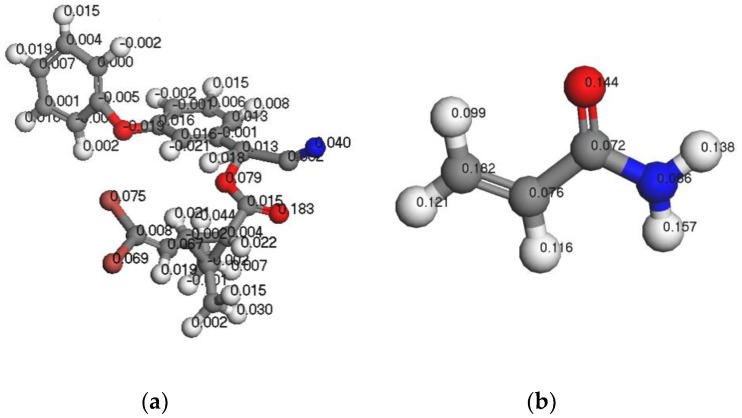
F (+) functions of DM (**a**) and AM (**b**).

The Fukui function can be used to qualitatively analyze the molecular reactive regions based on the Fukui frontier orbital theory. Thus, using the Fukui function, one can determine the active sites of the most stable complex formed by the template molecule and functional monomer. Among the various Fukui functions, the Fukui index for nucleophilic attack (Fukui (+)) and the Fukui index for free radical attack (Fukui (0)) indicate that the atoms are susceptible to nucleophilic agents and attack by free radicals, respectively. On the other hand, the Fukui index for electrophilic attack (Fukui) (−)) is reflective of the sensitivity of the atoms to attack by electrophiles. Figure 3, Figure 4 and Figure 5 show the Fukui functions of the template molecule, DM, and the functional monomer, AM.

As indicated in Figure 3, Figure 4 and Figure 5, the most vulnerable atoms in the DM molecule were found to be the carbonyl oxygen atoms (Fukui (−) = 0.212), followed by the adjacent oxygen atoms (Fukui (−) = 0.199). The atom most vulnerable to nucleophile attack was determined to be the carbonyl oxygen atom (Fukui (+) = 0.183), followed by the adjacent carbonyl oxygen atom (Fukui (+) = 0.079). The atom most vulnerable to free radical attack was found to be the carbonyl oxygen atom on the aromatic ring side chain (Fukui (0) = 0.159). In the case of AM, the most vulnerable atom is the O atom in –C=O (Fukui (−) = 0.393), followed by H9 in –NH (Fukui (−) = 0.097). Furthermore, the atom most vulnerable to free radical attack is the O atom in –C=O (Fukui (0) = 0.269), followed by H9 in –NH (Fukui (0) = 0.169). Finally, the atom most vulnerable to nucleophile attack is C (1) (Fukui (+) = 0.182), followed by H9 (Fukui (+) = 0.157) of –NH. By combining the results of the FMO analysis and the MEP simulations with the Fukui index calculation results, we confirm that if the DM and the AM form a stable prepolymer, the preferable action site is between the carbonyl oxygen atom in the DM and atom H9 in the AM.

### 3.2. H-NMR Analysis of a Prepolymerized System

The strengths of the hydrogen bonds formed between the template and monomer molecules can be investigated using ^1^H-NMR [30]. The electron density around a proton will decrease when it participates in an H-bond. This can cause the chemical shift of the proton to move downfield. Moreover, the larger the chemical shift, the stronger the interaction between the template and monomer. Here, ^1^H-NMR spectroscopy was used to investigate the interactions between DM and AM, to obtain information regarding the nature of such interactions based on the chemical shift of the related protons.

**Figure 6:**
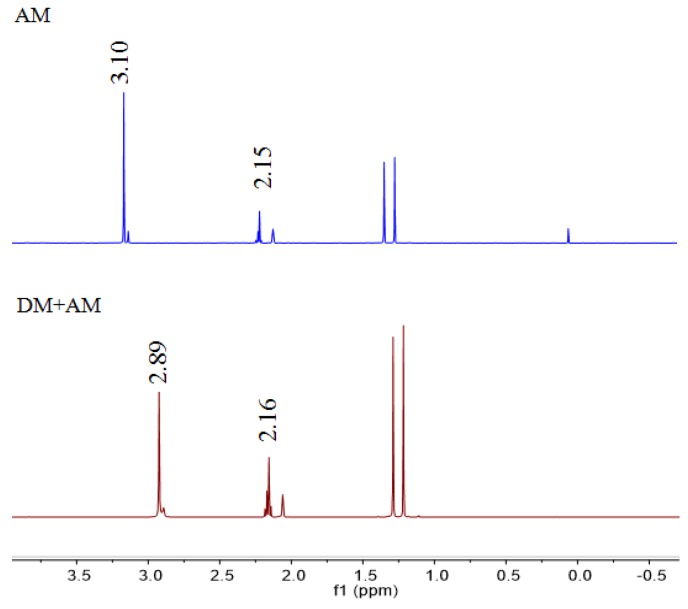
^1^H-NMR spectra of AM and DM–AM complex.

As can be seen in Figure 6, the peak related to the amino hydrogen atom of AM shifted downfield from 3.10 ppm to 2.89 ppm in the case of the DM–AM complex. A possible explanation for this result is that there is a hydrogen bond interaction between the carbonyl group of DM and the amino group of AM [31], which reduces the electron cloud density around the amino hydrogen atom of AM, resulting in the observed downfield shift. This would be indicative of the successful conjugation of DM with AM [32].

### 3.3. FTIR Analysis

**Figure 7:**
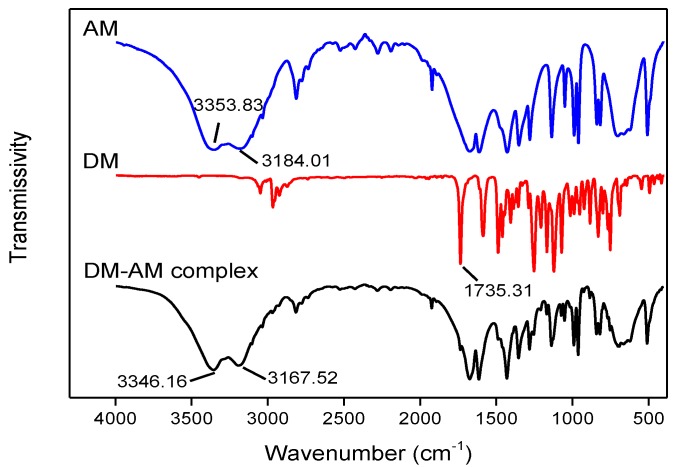
FT-IR spectra of AM, DM, and the DM–AM complex.

It is known that FTIR is a useful technique for identifying the incorporated functional groups and, in particular, quantifying the degree of polymerization and determining the types of polymerizable groups present in the reactive monomer [33]. The formation of hydrogen bonds can be discerned through FTIR analysis based on the shifts observed in the various peaks. Hence, to verify the simulation results, the FTIR spectra of DM, AM, and the DM–AM complex were determined. The results are shown in Figure 7.

In the spectrum of AM, the strong bands at 3354 and 3184 cm^−1^ can be attributed to the stretching vibrations of N–H in AM. Furthermore, the characteristic peak of C=O at 1735 cm^−1^ was also seen in the spectrum of DM. In addition, it can be seen that, compared to the pure AM, the N–H stretching peak of the DM–AM complex was shifted slightly to a shorter wavenumber (3354 to 3346 cm^−1^, 3184 to 3167 cm^−1^), indicating the formation of hydrogen bonds. This formation of hydrogen bonds affected the distribution of the electron cloud on the amino group of AM, which, in turn, resulted in a slight decrease in the frequency of the extension vibrations [34]. Therefore, the FTIR results confirmed that the C=O group of DM reacts with N–H in AM to form a hydrogen bond, which is the primary binding point between the template and the polymer.

### 3.4. Adsorption Thermodynamics

The thermodynamic parameters for the AM monomer and the adsorption of DM on MIPs at different temperatures (293, 303, 313, and 323 K) were calculated using Equations (1)−(5); the results are listed in Table 1. It can be seen from Table 1 that the ΔS of AM increased with the increasing temperature, implying that the disorder of the AM system increased and the stability decreased. The Δ*S* in the process of MIP adsorption template DM is much smaller than that of the AM monomer, indicating that the imprinting process is accompanied by a transformation from disorder to order due to the intermolecular interaction [35]. The ∆*H* and Δ*G* of MIPs were negative and their absolute values decreased with increasing temperature, confirming that the adsorption of the template molecules by the MIPs is a spontaneous exothermic process [36], with the process being less favorable at higher temperatures [37]. Moreover, based on the relationship between the thermodynamic parameters and the intermolecular binding force reported by Machicote and Zhao [38,39], one can surmise that when Δ*H* > 0 and Δ*S* > 0, the hydrophobic binding force is the primary binding force. When Δ*H* <0 and Δ*S* <0, hydrogen bonding is the main binding force. When Δ*H* <0 and Δ*S* > 0, the electrostatic force is the main binding force. In our results, both Δ*H* and Δ*S* are negative for the interaction between DM and the MIPs, so it can be speculated that hydrogen bonds play a critical role in the binding process.

### 3.5. The Effect of the Solvent on MIPs Molecular Recognition

In the molecularly imprinted polymer synthesis process, the solvent had a remarkable effect on the cavity shape and mechanical properties of MIPs. This is because the solvent can provide a porous structure for the imprinted molecular polymer to increase the speed of bonding the template molecule during recognition [29]. In this section, four solvents, n-hexane, chloroform, acetone, and acetonitrile, were selected to analyze the impact on the monomer and template.

**Figure 8:**
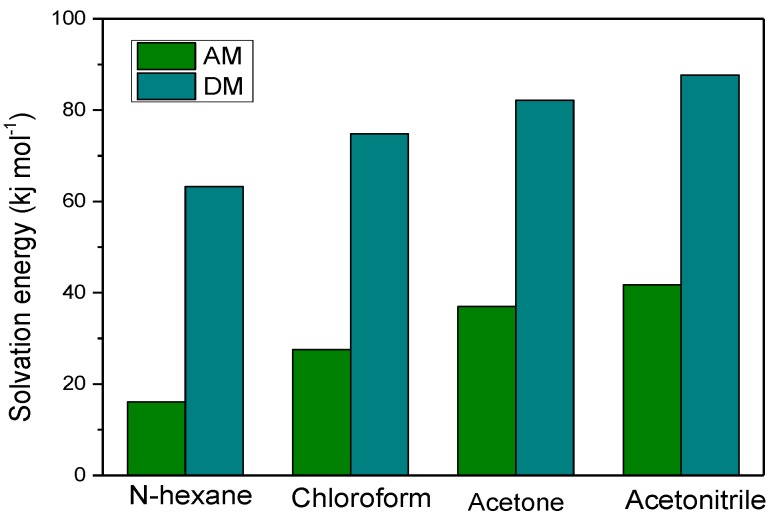
Solvation energy of DM and AM in different solvents.

As shown in Figure 8, the order of the solvation energy of AM and DM in different solvents is as follows, acetonitrile > acetone > chloroform > n-hexane, indicating that DM or AM has a stronger interaction with acetonitrile than with n-hexane and chloroform. The solvent molecules also act as a competitor to weaken the intermolecular interaction between the template molecule and the monomer. Therefore, when a solvent having a high solvation value is selected as a porogen, the molecular recognition effect of the obtained DM MIPs will be poor. Our previous studies also found that DM MIPs can be successfully synthesized using n-hexane, acetone, or chloroform as a solvent, but not acetonitrile. The adsorption capacity of the polymers synthesized with chloroform was higher than that of n-hexane (the adsorption capacity was 52.18 mg·g^−1^ and 47.46 mg·g^−1^, respectively). It is indicated that the solubility of the target molecule in the solvent should also be considered in practice. The weak polarity of the chloroform solvent can increase the solubility and accelerate the polymerization relative to the nonpolarity of n-hexane, which can allow the imprinted polymers to obtain a better molecular recognition effect.

### 3.6. Specific Adsorption Characteristics of DM MIPs

**Figure 9:**
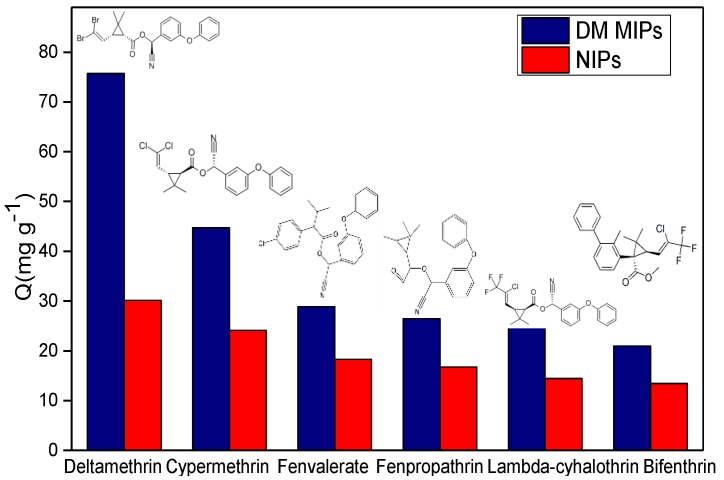
Specific adsorption capacities of MIPs and NIPs with respect to DM, cypermethrin, lambda-cyhalothrin, and bifenthrin.

To examine the accuracy of the theoretical calculations performed, the adsorption capacities of the synthesized DM MIPs and NIPs with respect to DM and its structural analogs (cypermethrin, fenvalerate, fenpropathrin, lambda-cyhalothrin, and bifenthrin) were measured. The results are shown in Figure 9.

Figure 9 shows that the DM MIPs exhibited the highest adsorption capacity for DM (75.72 mg g^−1^), which was 1.69 times higher than that for cypermethrin, 2.62 times higher than that for fenvalerate, 2.82 times higher than that for fenpropathrin, 3.06 times higher than that for lambda-cyhalothrin, and 3.61 times higher than that for bifenthrin. The structures of the five pyrethroid pesticides are similar to that of DM; however, the difference between them in terms of the two bromine atoms reduces their degree of matching with the three-dimensional pore structure of the fabricated DM MIPs, resulting in the observed low affinities. Of the five analogues tested, cypermethrin shows the highest degree of structural similarity with DM, while bifenthrin has the lowest. Thus, the results confirmed that the fabricated DM MIPs could differentiate between the DM template and the other molecules based on the shape, size, and spatial arrangement of the binding sites involved [40], which is accordance with the results of Mazouz et al. [41]. Furthermore, the adsorption capacities of the NIPs with respect to DM, cypermethrin, fenvalerate, fenpropathrin, lambda-cyhalothrin, and bifenthrin were lower than that of the DM MIPs, owing to a lack of the features mentioned above. Hence, the DM MIPs exhibited high selectivity towards DM.

### 3.7. Selective Extraction of DM in Different Tea Samples

In order to investigate the potential of DM MIPs for selective extraction of target analytes from complex matrices, different tea samples (black tea, green tea, tieguanyin tea, and black tea beverages) containing 15 mg L^−1^ DM were applied to detect the separation ability of DM MIPs using the molecularly imprinted solid phase extraction column (MISPE) protocol. The recovery rates of DM in different teas obtained by MISPE extraction are portrayed in Table 2. The results show that the recovery rate of deltamethrin in different teas was 83.68–107.55% (RSD = 0.35–2.13%). This is not very different from the recovery rate of hydrochlorothiazide (HCT) in serum and pharmaceutical samples designed and synthesized by Nezhadali et al. through a DFT-based computational approach (the HCT recovery rate rang was 99.5–105.75%) [42]. This proved that the molecularly imprinted polymer calculations predicted with computer simulation technology can be successfully applied to the actual environment for sample purification and enrichment.

## 4. Conclusions

In the present study, the intermolecular interactions between a template molecule (DM) and a functional monomer (AM) were investigated through molecular simulations performed based on the DFT-B3LYP/GGA level of theory. The HOMO–LUMO energy gap and MEP were analyzed to determine the chemical potential and electronegativity values of the molecules, which are further used to predict the practical reaction regions. Moreover, the results of Fukui function calculations confirmed that the site for the binding of the template to the monomer was mainly located on atom O19 of DM and atom H9 of AM. The ^1^H-NMR and FTIR analyses were performed to determine the nature of the hydrogen bond interactions between DM and AM. Thermodynamic analysis showed that the adsorption of DM by the MIPs is an exothermic process and that the hydrogen bonding force is the primary force involved in the binding process. Specific adsorption tests showed that the fabricated MIPs exhibited higher selectivity towards DM as compared to its structural analogues. More than 83.68% of the DM in various teas could be recovered via solid-phase extraction using the DM MIPs as the column filler. This study can provide a more detailed view of the intermolecular interactions that occur between DM molecules and their functional counterparts, providing a theoretical basis for the fabrication of MIPs for the selective detection of DM in complex matrices.

## Figures and Tables

**Table 1:** Thermodynamic parameters for AM and adsorption of DM on synthesized MIPs.

	Δ*H*/kJ mol^−1^	Δ*S*/J mol^−1^ K				Δ*G*/kJ mol^−1^			
		293 K	303 K	313 K	323 K	293 K	303 K	313 K	323 K
**AM Monomer**	185.351	0.627	0.606	0.586	0.568	−1.593	−1.677	−1.681	1.686
**DM MIPs**	−61.00	188.9	188.0	185.1	181.2	−5.655	−4.046	−3.052	2.489

**Table 2:** The recovery rate of DM in different tea samples.

Tea type	Recovery (%)	RSD (%)
Black tea	83.68	2.13
Green tea	107.55	0.98
Tieguanyin	94.38	1.79
Black tea beverage	85.82	0.35

## Appendix A

The experimental procedure of the adsorption of DM in different teas by DM MIPs solid-phase extraction. First, weigh 3.0 g of ground tea leaves (black, green or Tieguanyin teas) in a 500 mL Erlenmeyer flask and added 450 mL of boiling distilled water. The mixture was shaken vigorously at 5 min intervals for 45 min, then filtered. The liquid phase was transferred to a 500 mL volumetric flask and allowed to cool to room temperature, after which distilled water was added to bring the total volume to 500 mL. Black tea beverage can be purchased in the market as a tea soup.Then, a solid phase extraction column with a polypropylene shell was filled with 0.100 g of the DM MIPs using the dry packing method. The MIPs were activated by the addition of 15 mL of water, then loaded with a tea sample containing 15 mg L^−1^ DM. The column packing was subsequently washed with 20% methanol in water, followed by elution with 10 mL of methanol at a flow rate of 1 mL min^−1^. The eluent was collected and the absorbance of this solution was determined at 220 nm using a UV-vis spectrophotometer, following which the DM concentration was calculated based on a standard calibration curve.
